# Towards more accurate HIV testing in sub-Saharan Africa: a multi-site evaluation of HIV RDTs and risk factors for false positives

**DOI:** 10.7448/IAS.20.1.21345

**Published:** 2017-03-24

**Authors:** Cara S Kosack, Anne-Laure Page, Greet Beelaert, Tumwesigye Benson, Aboubacar Savane, Anne Ng’ang’a, Bita Andre, Jean-Paul BN Zahinda, Leslie Shanks, Katrien Fransen

**Affiliations:** ^a^ Médecins sans Frontières, Diagnostic Network, Amsterdam, Netherlands; ^b^ Epicentre, Epidemiology and Population Health Department, Paris, France; ^c^ Institute of Tropical Medicine, AIDS Reference Laboratory, Antwerp, Belgium; ^d^ Ministry of Health Uganda, STD/AIDS Control Program, Kampala, Uganda; ^e^ Ministry of Health Guinea, Laboratoire National de Reference, Conakry, Guinea; ^f^ Ministry of Health Kenya, National AIDS and Sexually Transmitted Infections Control Programme, Nairobi, Kenya; ^g^ Ministry of Health Cameroon, Regional Delegation of Public Health for the Littoral Region, Yaoundé, Cameroon; ^h^ Ministry of Health Democratic Republic of Congo, Programme National de Lutte contre le Sida et les IST (PNLS), Bukavu, Democratic Republic of Congo; ^i^ Public Health Departement, Médecins sans Frontières, Amsterdam, Netherlands

**Keywords:** HIV, diagnostic, test, RDT, false positive

## Abstract

**Introduction**: Although individual HIV rapid diagnostic tests (RDTs) show good performance in evaluations conducted by WHO, reports from several African countries highlight potentially significant performance issues. Despite widespread use of RDTs for HIV diagnosis in resource-constrained settings, there has been no systematic, head-to-head evaluation of their accuracy with specimens from diverse settings across sub-Saharan Africa. We conducted a standardized, centralized evaluation of eight HIV RDTs and two simple confirmatory assays at a WHO collaborating centre for evaluation of HIV diagnostics using specimens from six sites in five sub-Saharan African countries.

**Methods**: Specimens were transported to the Institute of Tropical Medicine (ITM), Antwerp, Belgium for testing. The tests were evaluated by comparing their results to a state-of-the-art reference algorithm to estimate sensitivity, specificity and predictive values.

**Results**: 2785 samples collected from August 2011 to January 2015 were tested at ITM. All RDTs showed very high sensitivity, from 98.8% for First Response HIV Card Test 1–2.0 to 100% for Determine HIV 1/2, Genie Fast, SD Bioline HIV 1/2 3.0 and INSTI HIV-1/HIV-2 Antibody Test kit. Specificity ranged from 90.4% for First Response to 99.7% for HIV 1/2 STAT-PAK with wide variation based on the geographical origin of specimens. Multivariate analysis showed several factors were associated with false-positive results, including gender, provider-initiated testing and the geographical origin of specimens. For simple confirmatory assays, the total sensitivity and specificity was 100% and 98.8% for ImmunoComb II HIV 12 CombFirm (ImmunoComb) and 99.7% and 98.4% for Geenius HIV 1/2 with indeterminate rates of 8.9% and 9.4%.

**Conclusions**: In this first systematic head-to-head evaluation of the most widely used RDTs, individual RDTs performed more poorly than in the WHO evaluations: only one test met the recommended thresholds for RDTs of ≥99% sensitivity and ≥98% specificity. By performing all tests in a centralized setting, we show that these differences in performance cannot be attributed to study procedure, end-user variation, storage conditions, or other methodological factors. These results highlight the existence of geographical and population differences in individual HIV RDT performance and underscore the challenges of designing locally validated algorithms that meet the latest WHO-recommended thresholds.

## Introduction

HIV rapid diagnostic tests (RDTs) are the main diagnostic tool for HIV screening and diagnosis in resource-constrained settings [1]. Simple and fast, they require little or no equipment, and provide results usually within 20 min. Most RDTs involve very few manipulation steps, can be read visually, and can be stored at ambient temperature. At a price per test of US$ 1–2, RDTs are ideal for use in settings without the infrastructure or expertise to support the use of more complex techniques.

Given the potential for severe psychological and social impacts of HIV misdiagnosis, it is imperative that HIV diagnosis is highly sensitive and specific. HIV misdiagnosis has been a problem in some Médecins Sans Frontières (MSF) programmes in sub-Saharan Africa where HIV care is provided in partnership with local Ministries of Health [2,3]. In addition to the psychological trauma a misdiagnosis can induce in the individual patient, who may inappropriately have been initiated on treatment that is both costly and potentially harmful, there is also the considerable programmatic impact of false positives, which siphon off scarce resources and may undermine client-patient confidence in the testing [4,5].

World Health Organization (WHO) guidelines for HIV testing and counselling recommend an algorithm consisting of 2–3 RDTs chosen on the basis of their performance (clinical sensitivity ≥99% and clinical specificity ≥98% for the first-line assay, and ≥99% for the second line assay), operational characteristics and local evaluation results, among other factors [1].

The latest WHO evaluations of single HIV RDTs reported highly sensitive and specific results, with most tests exceeding the recommended thresholds for performance [6,7]. However, the results of studies of RDT accuracy at laboratory and field level are more varied than they are for HIV testing algorithms [8–19].

Despite the continuing widespread use of RDTs for HIV diagnosis in resource-constrained settings, there has been no systematic, head-to-head evaluation of their accuracy with specimens from diverse settings across sub-Saharan Africa.

We report here the results of a standardized, centralized evaluation of eight HIV RDTs and two simple confirmatory assays at a WHO collaborating centre for evaluation of HIV diagnostics using specimens collected from six sites in five sub-Saharan African countries. Algorithms will be elucidated and discussed in a separate publication.

## Methods

### Study setting

This study was carried out at six public health care clinics and hospitals in sub-Saharan Africa where Médecins Sans Frontières (MSF) supports health care activities: (1) Centre Communautaire Matam in Conakry, Guinea, (2) Madi Opei Clinic and Kitgum Matidi Clinic in Kitgum, Uganda, (3) Homa Bay District Hospital in Homa Bay, Kenya, (4) Arua District Hospital in Arua, Uganda, (5) Nylon Hospital in Doula, Cameroun and (6) Baraka Hospital in Baraka, South-Kivu, DRC. The six sites were selected from among MSF-supported HIV testing and counselling (HTC) sites to represent geographical diversity and a range of characteristics (urban and rural, voluntary and provider-initiated testing, different HIV prevalence). The HIV national reference laboratory at the Institute of Tropical Medicine (ITM, Antwerp, Belgium) served as the central laboratory for this study.

### Study design and sample size

This was a multi-centre evaluation of the diagnostic accuracy of eight individual HIV RDTs and two simple HIV confirmatory assays on the following measures: sensitivity, specificity and predictive values.

At least 200 positive and 200 negative samples from study participants were collected for evaluation at each study site [20]. The sample size was calculated based on the assumption that both sensitivity and specificity must be 98% in order to provide a 95% confidence interval of less than ±2% for both sensitivity and specificity.

The prevalence of HIV positives among the suspects tested at each study site was known. If it was ≥40%, we collected all specimens consecutively and calculated the total sample size based on the prevalence to obtain at least 200 HIV-positive and 200 HIV-negative samples and increased the calculated sample size by 10% to account for losses and/or problems in shipment.

If the prevalence of positive results was below 40%, we obtained a subsample of positive and negative specimens. Conservatively assuming 10% misclassification, we collected a sub-sample of 220 positive and 220 negative samples based on the on-site algorithm result. All samples with an inconclusive result were included. For this sampling strategy, we first included consecutively all clients, regardless of their results. Once the sample size for negative clients was reached, we stopped including HIV-negative clients (based on their on-site results) and included all clients diagnosed as HIV positive or inconclusive, for example, RDT1 positive and RDT2 negative, based on the on-site algorithm.

### Study population

Clients ≥5 years of age who attended any of the participating HIV testing and counselling (HTC) centres and for whom written informed consent was provided by the client or legal guardian were included in the study. Upon enrolment, clients were offered HTC in accordance with site-specific procedures and testing algorithms. Exclusion criteria were: withdrawal of consent; inability to obtain a venous blood sample or insufficient blood; and current or past enrolment on anti-retroviral treatment.

### Sample collection, storage and transportation

Venous EDTA blood was collected by the study nurse or laboratory technician. The EDTA blood samples were centrifuged, aliquoted and stored at −20 °C until being transported at 2–8 °C to the central laboratory (ITM) in Belgium. The storage temperature of freezers was monitored daily and a temperature recording system was used during transportation. At ITM, samples were immediately tested using the reference algorithm and remaining plasma samples were aliquoted further and stored a −20 °C until testing of RDTs.

### Reference method for HIV diagnosis

**Figure 1.**
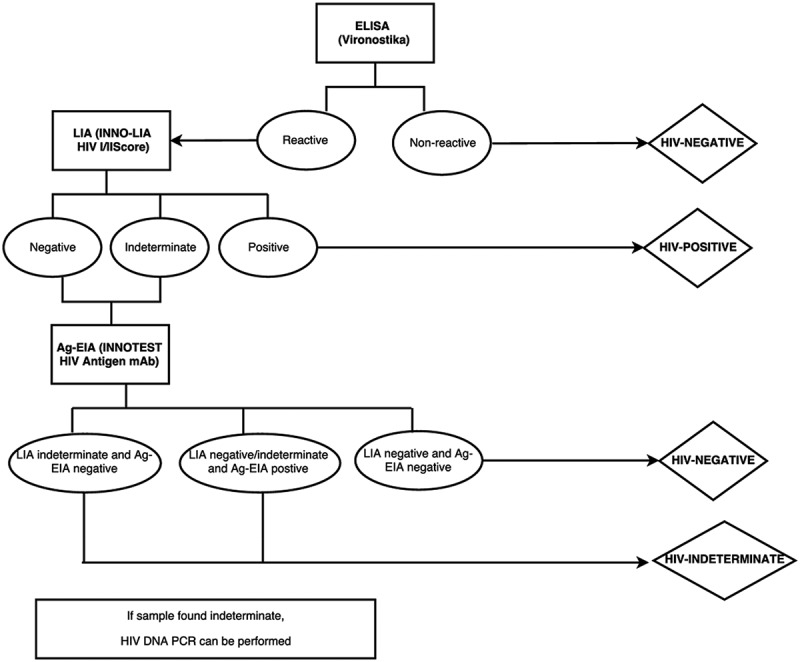
Reference algorithm at the AIDS reference laboratory at the Institute of Tropical Medicine, Antwerp, Belgium.

Clients’ status was determined by using the reference standard algorithm at the AIDS reference laboratory at ITM, Antwerp, Belgium (Figure 1) on collected plasma samples. All samples were tested by a fourth generation ELISA (Vironostika® HIV Uni-Form II Ag/Ab, bioMérieux, France) and all reactive samples were confirmed by a Line-Immunoassay (LIA, i.e. INNO-LIA™ HIV I/II Score, Innogenetics NV, Ghent, Belgium). Samples with a negative or indeterminate LIA were tested with an antigen-enzyme-immunoassay (Ag-EIA, i.e. INNOTEST HIV Antigen mAb, Innogenetics NV, Ghent, Belgium) to confirm acute infections. In the event that the LIA could not differentiate between HIV-1 and HIV-2, we used an in-house DNA PCR.

### HIV RDT

The following eight HIV RDTs were tested at ITM on all plasma samples collected from the six study sites. Determine HIV-1/2 (Determine, Alere, USA; #7D2347), Uni-Gold HIV (Uni-Gold, Trinity Biotech, Ireland; #1206502), Genie Fast HIV 1/2 (Genie Fast, BioRad Laboratories, USA; #72330), Vikia HIV 1/2 (Vikia, bioMérieux, France; #31 112), HIV 1/2 STAT-PAK (STAT-PAK, Chembio, USA; #HIV101), INSTI HIV-1/HIV-2 Antibody Test (INSTI, bioLytical, Canada; # 90–1021), SD Bioline HIV 1/2 3.0 (SD Bioline, Standard Diagnostics, Korea; #03FK10), and First Response HIV Card Test1–2.O (First Response, PMC, India; # 05FRC30). Each test was read by two laboratory technicians who were blinded to each other’s result. If a reader disagreed, a third reader acted as tiebreaker.

All but one of the RDTs is prequalified by the WHO [21], and the one exception, Genie Fast, has been submitted for prequalification [22].

The two simple confirmatory assays evaluated were: ImmunoComb II HIV 1&2 CombFirm (ImmunoComb, Orgenics, Alere, Israel; #60434002) and Geenius HIV 1/2 confirmatory assay (Geenius, Bio-Rad, USA; #72460). The latter was interpreted both by using the Geenius reader system and the technician’s naked eye. Though neither of the simple confirmatory assays is WHO prequalified, the Geenius assay has been submitted for prequalification [22].

**Figure 2.**
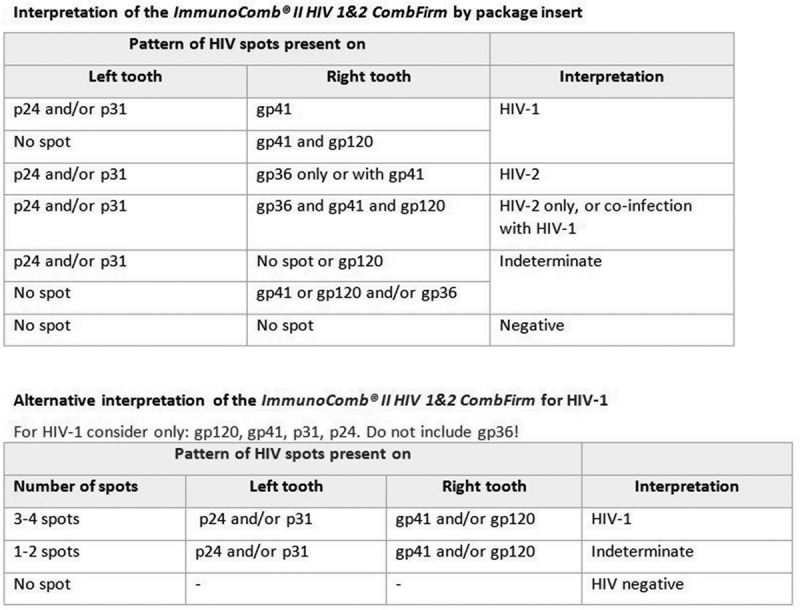
Manufacturer’s and alternative interpretation of the ImmunoComb II HIV 1&2 CombFirm (Orgenics, Alere, Israel).

Tests were performed and interpreted according to the manufacturer’s instructions. An additional analysis was performed with the ImmunoComb using an alternate interpretation based on the strict criteria used in an earlier evaluation (Figure 2) [2].

All tests were read by two laboratory technicians who were blinded to each other’s interpretation and to the client’s HIV status. If the two readers disagreed, a third reader acted as tie-breaker. Band intensity was recorded by the two readers and graded from 1 to 3 (1 = weak line, 2 = medium strength line, 3 = strong line).

### Statistical analysis

Data were analyzed using Stata version 13.1 (StataCorp, College Station, Texas, USA).

We estimated the sensitivity, specificity and predictive value for each RDT and simple confirmatory assay by comparing the results of these tests performed at ITM to the results of the reference standard. The analysis was weighted to adjust for the sampling strategy, which under-represented negative samples. For each participant, the weight was calculated as the inverse of the probability of inclusion in the study. For the total adjusted estimates, the weights were normalized to ensure equal representation of each site. Weighted proportions (e.g. weighted proportion of RDT reactive among all true positives by the reference standard for sensitivity) were calculated using the svy survey prefix command in Stata.

To measure inter-reader reliability, the level of concordance between results reported by the two laboratory technicians independently reading the test was evaluated using the kappa coefficient. A Kappa value ≥80% was considered very good agreement.

For each rapid test, factors associated with false positivity were analyzed using logistic regression with age, gender, inclusion site, entry mode and comorbidity included as co-variates.

### Ethics

The study was approved by the MSF Ethics Review Board and the Ethics Committee of the five countries where the study took place.

## Results

### Characteristics of the study population

From August 2011 to January 2015, a total of 2785 samples were collected at the six HTC sites and tested at the central laboratory (Table 1), with 437–500 samples collected per study site. Of the total 2785 samples, 1474 were found to be HIV negative and 1306 HIV positive (including one positive for HIV-2) by the reference algorithm (Figure 1). Three samples with indeterminate results and two classified as acute infections were excluded from the analysis.

**Table 1.** Demographic and clinical characteristics by study site

	Guinea, Conakry		Uganda, Kitgum	Uganda, Arua	Kenya, Homa Bay	Cameroun, Douala	DRC, Baraka	Total
**Total tested at site**	HTC	ANC						
Total, *n*	793	1240	3159	2971	1003	1239	3610	14015
Positive, *n* (%)	505 (63.7)	69 (5.5)	332 (10.5)	386 (13.0)	372 (37.1)	396 (32.0)	288 (8.0)	2348 (16.8)
Negative, *n* (%)	278 (35.0)	1169 (94.3)	2827 (89.5)	2585 (87.0)	617 (61.5)	826 (66.7)	3252 (90.1)	11554 (82.4)
Indeterminates, *n* (%)	10 (1.3)	2 (0.2)	0 (0)	0 (0)	14 (1.4)	17 (1.4)	70 (1.9)	113 (0.8)
**Included in the study based on HIV status tested at site**								
Total, *n*	341	105	437	443	500	462	497	2785
Positive, *n* (%)	220 (64.5)	2 (1.9)	217 (49.7)	212 (47.9)	223 (44.6)	222 (48.1)	221 (44.5)	1317 (47.3)
Negative, *n* (%)	117 (34.3)	103 (98.1)	220 (50.3)	231 (52.1)	277 (55.4)	230 (49.8)	220 (44.2)	1398 (50.2)
Indeterminates, *n* (%)	4 (1.2)	0 (0)	0 (0)	0 (0)	0 (0)	10 (2.2)	56 (11.3)	70 (2.5)
**Entry mode**								
Voluntary testing, *n* (%)	0 (0)		323 (73.9)	443 (100)	459 (91.8)	211 (45.7)	187 (37.8)	1623
Spouse, *n* (%)	238 (53.4)		20 (4.6)	0 (0)	0 (0)	251 (54.3)	10 (2.0)	519
Referred – TB clinic, *n* (%)	57 (12.8)		2 (0.5)	0 (0)	21 (4.2)	0 (0)	3 (0.6)	83
Referred – IPD, *n* (%)	33 (7.4)		0 (0)	0 (0)	0 (0)	0 (0)	297 (59.8)	330
Referred – OPD, *n* (%)	13 (2.9)		33 (7.5)	0 (0)	0 (0)	0 (0)	0 (0)	46
ANC, *n* (%)	105 (23.3)		54 (12.4)	0 (0)	20 (4.0)	0 (0)	0 (0)	179
Other, *n* (%)	0 (0)		5 (1.1)	0 (0)	0 (0)	0 (0)	0 (0)	5
**Age and gender**								
Median age (IQR)	29 (22–39)		30 (24–39)	29 (23–37)	30 (23–40)	31 (25–41)	30 (23–39)	30 (24–39)
Males, *n* (%)	132 (29.6)		176 (40.3)	213 (48.2)	201 (40.2)	163 (35.3)	177 (35.6)	1062 (38.1)


HTC = HIV testing and counselling, ANC = antenatal care, TB = tuberculosis, IPD = in-patient department; OPD = out-patient department.

Most study participants were females (61.9%). The median age of study participants was 30 (IQR: 24–39). Most participants presented for testing at the HTC facility voluntarily, or were referred by their spouse, with variations among sites (Table 1).

### Diagnostic accuracy of the HIV RDTs

Adjusted (weighted) sensitivities ranged from 96.2% to 100% with specimens from different study sites (Table 2). Adjusted sensitivities <99% were found for four tests (Uni-Gold, Vikia, STAT-PAK and First Response) using specimens from Kitgum; and for the First Response test using specimens from Douala (97.7%) and Baraka (96.8%). The First Response was the only RDT with an overall (total) adjusted sensitivity <99% (Table 2). Unadjusted (unweighted/crude) sensitivities are shown in Additional File 1.

**Table 2.** Weighted diagnostic accuracy of HIV RDTs

	Guinea, Conakry	Uganda, Kitgum	Uganda, Arua	Kenya, Homa Bay	Cameroun, Douala	DRC, Baraka	Total
**Sensitivity; 95% CI**							
Determine	100; 98.3–100	100; 98.3–100	100; 98.3–100	100; 98.3–100	100; 98.3–100	100; 98.3–100	100; 99.7–100
Uni-Gold	100; 98.3–100	96.2; 77.9–99.5	100; 98.3–100	99.6; 96.8–99.9	100; 98.3–100	99.6; 96.8–99.9	99.5; 98.1–99.9
Genie Fast	100; 98.3–100	100; 98.3–100	100; 98.3–100	100; 98.3–100	100; 98.3–100	99.6; 96.8–99.9	100; 99.8–100
Vikia	100; 98.3–100	96.2; 77.9–99.5	100; 98.3–100	100; 98.3–100	99.5; 96.7–99.7	99.6; 96.8–99.9	99.6; 98.1–99.9
STAT-PAK	100; 98.3–100	96.2; 77.9–99.5	100; 98.3–100	99.6; 96.8–99.9	99.5; 96.7–99.7	100; 98.3–100	99.5; 98.1–99.8
INSTI	100; 98.3–100	100; 98.3–100	100; 98.3–100	100; 98.3–100	100; 98.3–100	100; 98.3–100	100; 99.7–100
SD Bioline	100; 98.3–100	100; 98.3–100	99.6; 97.0–99.9	100; 98.3–100	100; 98.3–100	100; 98.3–100	100; 99.7–100
First Response	100; 98.3–100	96.2; 77.9–99.5	99.1; 96.6–99.8	100; 98.3–100	97.7; 94.5–99.0	96.8; 93.5–98.5	98.8; 97.7–99.4
**Specificity; 95% CI**							
Determine	99.0; 97.7–99.6	93.1; 88.8–95.8	94.4; 90.6–96.8	94.4; 91.0–96.5	92.4; 88.8–94.9	91.9; 87.8–94.7	93.9; 92.6–95.0
Uni-Gold	99.2; 94.7–99.9	98.2; 95.2–99.3	96.9; 93.7–98.5	99.0; 96.9–99.7	97.8; 94.7–98.8	96.5; 93.3–98.2	97.8; 96.9–98.5
Genie Fast	97.5; 93.9–99.0	93.5; 89.4–96.1	88.0; 83.1–91.6	96.5; 93.5–98.1	95.2; 91.7–97.2	95.7; 92.5–97.6	94.1; 92.7–95.3
Vikia	99.0; 97.7–99.6	96.8; 93.4–98.5	96.3; 93.0–98.1	96.9; 94.1–98.4	97.5; 95.1–98.8	96.8; 93.8–98.4	97.2; 96.2–97.9
STAT-PAK	100; 98.3–100	100; 98.3–100	99.9; 99.5–100	98.6; 96.2–99.5	99.6; 98.3–99.9	99.9; 99.7–100	99.7; 99.4–99.9
INSTI	98.0; 94.1–99.3	96.3; 92.8–98.2	90.4; 85.8–93.6	96.8; 94.0–98.3	84.9; 79.8–88.9	80.4; 74.8–85.0	90.6; 88.9–92.0
SD Bioline	99.7; 98.7–99.9	98.6; 95.8–99.5	95.6; 92.0–97.6	96.8; 94.0–98.4	98.7; 96.2–99.6	96.6; 93.4–98.3	97.6; 96.5–98.4
First Response	98.0; 94.1–99.3	90.4; 85.7–93.6	77.0; 71.1–82.0	85.3; 80.6–89.0	99.8; 98.5–100	93.2; 89.1–95.8	90.4; 88.7–91.8
**Positive predictive value; 95% CI**							
Determine	97.5; 94.4–98.9	63.4; 50.6–74.5	72.3; 60.5–81.7	91.6; 86.7–94.8	85.4; 79.2–90.0	51.8; 40.5–62.9	82.0; 78.6–85.0
Uni-Gold	98.1; 87.5–99.7	86.3; 70.0–94.5	84.4; 71.6–92.0	98.4; 95.1–99.5	94.7; 89.1–97.5	71.2; 55.6–83.0	92.6; 89.7–94.7
Genie Fast	94.0; 86.0–97.5	65.0; 51.9–76.2	57.3; 47.3–66.8	94.5; 90.1–97.0	90.3; 83.9–94.3	66.8; 52.5–78.5	82.5; 78.8–85.6
Vikia	97.5; 94.4–98.9	78.3; 62.8–88.5	82.0; 69.5–90.1	95.2; 91.0–97.5	94.7; 89.7–97.3	73.0; 57.5–84.3	90.7; 87.8–93.0
STAT-PAK	100; 98.4–100	100; 98.3–100	99.6; 97.0–99.9	97.7; 94.0–99.1	99.1; 96.3–99.8	98.7; 96.1–99.6	99.0; 97.9–99.5
INSTI	95.1; 86.4–98.3	76.6; 61.7–97.0	61.9; 51.2–71.5	94.5; 90.1–97.0	74.7; 67.5–80.8	30.7; 24.3–37.8	74.6; 71.1–77.9
SD Bioline	99.2; 96.7–99.8	89.5; 73.6–96.3	79.0; 66.5–87.8	95.1; 90.8–97.4	97.3; 91.9–99.1	71.7 55.9–83.5	92.0; 88.7–94.4
First Response	95.1; 86.4–98.3	54.5; 43.3–65.2	41.8; 34.3–49.6	80.6; 74.9–85.3	99.5; 96.7–99.9	55.2; 42.4–67.3	73.9; 70.3–77.3
**Negative predictive value; 95% CI**							
Determine	100; 98.3–100	100; 98.2–100	100; 98.3–100	100; 98.6–100	100; 98.3–100	100; 98.4–100	100; 99.7–100
Uni-Gold	100; 98.4–100	99.5; 96.8–99.9	100; 98.3–100	99.7; 98.1–100	100; 98.5–100	100; 99.7–100	99.9; 99.5–100
Genie Fast	100; 98.3–100	100; 98.2–100	100; 98.2–100	100; 98.6–100	100; 98.4–100	100; 99.7–100	100; 99.9–100
Vikia	100; 98.3–100	99.5; 96.7–99.9	100; 98.3–100	100; 98.6–100	99.8; 98.5–100	100; 99.7–100	99.9; 99.5–100
STAT-PAK	100; 98.4–100	99.5; 96.8–99.9	100; 98.4–100	99.7; 98.1–100	99.8; 98.5–100	100; 98.7–100	99.9; 99.5–100
INSTI	100; 98.3–100	100; 98.3–100	100; 98.2–100	100; 98.6–100	100; 98.3–100	100; 98.3–100	100; 99.7–100
SD Bioline	100; 98.4–100	100; 98.3–100	99.9; 99.5–100	100; 98.6–100	100; 98.5–100	100; 98.6–100	100; 99.9–100
First Response	100; 98.3–100	99.5; 96.5–99.9	99.8; 99.3–100	100; 98.4–100	99.0; 97.5–99.6	99.7; 99.4–99.9	99.7; 99.3–99.9

Adjusted specificities across the six sites varied from 77.0% for First Response on specimens from Kitgum to 100% for STAT-PAK on specimens from Conakry and Kitgum (Table 2). The INSTI and the First Response test had the lowest overall adjusted specificities (<90%), while STAT-PAK was the only RDT with an adjusted total specificity >98% (Table 2).

### HIV RDTs differentiating HIV-1 and −2

Only the SD Bioline and First Response tests could distinguish HIV-1 and HIV-2 by a separate reaction line. Since only one participant was infected with HIV-2, we could not assess the tests’ sensitivity for HIV-2, only their specificity, which was 89.8% (95% CI: 88.6-90.9; 2490/2774) for SD Bioline and 96.1% (95% CI: 95.3–96.8; 2665/2774) for First Response (Table 3).

**Table 3.** Comparison of SD Bioline HIV 1/2 3.0 and first response HIV Card Test 1-2.0 with the reference method results, including differentiation between HIV-1 and HIV-2 (*N* = 2780)

		Results of the reference test				
		Negative	HIV-1	HIV-2	HIV^a^	Total
SD Bioline	Non-reactive	1431	1	0	0	1432
	HIV-1	31	1027	0	1	1059
	HIV-2	10	4	1	0	15
	HIV-1 & HIV-2	2	268	0	4	274
First Response	Non-reactive	1332	15	0	0	1347
	HIV-1	119	1199	0	2	1320
	HIV-2	1	0	1	0	2
	HIV-1 & HIV-2	22	86	0	3	111
	Total	1474	1300	1	5	2780

^a^Specimens could not be differentiated because the dried blood spot sample for PCR was not collected.

### Band intensity and inter-reader agreement

The proportion of weak bands (intensity = 1) read by each of the readers is shown in Table 4. Weak bands were seen only with the SD Bioline and First Response tests and represented up to half of the total reactive HIV-2 lines (Tables 3 and 4).

**Table 4.** Proportion of weak bands (line intensity = 1) per RDT (*N* = 2780)

	Reader A			Reader B		
	*n*	Proportion (%)		*n*	Proportion (%)	
		overall	positives		overall	positives
Determine	0	0	0	0	0	0
Uni-Gold	0	0	0	1	0	0
Genie Fast	0	0	0	0	0	0
Vikia	0	0	0	0	0	0
STAT-PAK	0	0	0	0	0	0
INSTI	0	0	0	0	0	0
SD Bioline – line 1	29	1.0	2.2	29	1.0	2.2
SD Bioline – line 2	265	9.5	46.7	268	9.6	47.8
First Response – line 1	262	9.4	18.2	237	8.5	16.5
First Response – line 2	66	2.4	51.2	54	1.9	42.5

Very good inter-reader agreement was found for all HIV RDTs, with kappa coefficients ranging from 98% to 100% (Table 5). The Vikia and STAT-PAK tests showed no disagreement between readers. The agreements for the simple confirmatory tests were lower than for the RDTs (Table 5).

**Table 5.** Inter-reader agreement (kappa) and absolute number of disagreements for all RDTs and simple confirmatory assays (*n* = 2785)

	Number of disagreements	Kappa
**Determine**	8	99.4
**Uni-Gold**	2	99.9
**Genie Fast**	5	99.6
**Vikia**	0	100.0
**STAT-PAK**	0	100.0
**INSTI**	14	99.0
**SD Bioline**	5	99.6
**First Response**	28	98.0
**ImmunoComb II HIV 1&2 CombFirm**	51	95.2
p24	25	99.1
p31	58	97.9
gp120	16	99.4
gp41	6	99.8
gp36	12	99.6
**Geenius HIV 1/2 confirmatory assay**	85	96.9
gp36	8	99.7
gp140	83	97.0
p31	73	97.4
gp160	5	99.8
p24	67	97.7
gp41	18	99.4
		

### Diagnostic accuracy of the simple HIV confirmatory assays

The total adjusted sensitivity of both simple confirmatory assays was close to 100% (Table 6). The specificity of the ImmunoComb increased from 98.9% seen with the manufacturer’s recommended interpretation to 99.4% when using the alternative interpretation criteria [18], while the rate of indeterminate results increased from 8.9% to 9.8%. The specificity of the Geenius assay varied from 97.6% to 98.3% for visual versus automated reading with similar rates of indeterminate results for visual reading (9.2%) and automated reading (9.4%). Overall, measurement with the automated reader was as accurate or more than with the naked eye (Table 6).

**Table 6.** Weighted/adjusted diagnostic accuracy of two simple confirmatory assays

	Guinea, Conakry	Uganda, Kitgum	Uganda, Arua	Kenya, Homa Bay	Cameroun, Douala	DRC, Baraka	Total
**ImmunoComb II HIV 1&2 CombFirm – interpretation according to manufacturer**							
Sensitivity; 95% CI	100; 98.3–100	100; 98.3–100	100; 98.3–100	100; 98.3–100	100; 98.3–100	100; 98.3–100	100; 99.7–100
Specificity; 95% CI	99.7; 98.6–99.9	98.3; 94.9–99.5	97.7; 94.1–99.1	100; 98.4–100	99.1; 96.6–99.7	98.5; 95.8–99.5	98.8; 98.0–99.3
PPV; 95% CI	99.2; 96.7–99.8	89.4; 72.8–96.4	90.1; 77.6–96.0	100; 98.4–100	98.2; 94.1–99.5	88.3; 71.5–95.7	96.5; 94.3–97.9
NPV; 95% CI	100; 98.3–100	100; 98.3–100	100; 98.3–100	100; 98.4–100	100; 98.3–100	100; 98.2–100	100; 99.7–100
**ImmunoComb II HIV 1&2 CombFirm – alternative interpretation**							
Sensitivity; 95% CI	100; 98.3–100	100; 98.3–100	100; 98.3–100	100; 98.4–100	100; 98.3–100	100; 98.3–100	100; 99.7–100
Specificity; 95% CI	99.7; 98.6–99.9	98.3; 94.9–99.5	99.3; 96.4–99.9	100; 98.4–100	100; 98.3–100	99.9; 99.7–100	99.5; 98.9–99.8
PPV; 95% CI	99.2; 96.7–99.8	89.2; 72.5–96.3	97.0; 85.1–99.5	100; 98.4–100	100; 98.3–100	99.6; 97.0–100	98.5; 96.7–99.4
NPV; 95% CI	100; 98.3–100	100; 98.3–100	100; 98.3–100	100; 98.4–100	100; 98.3–100	100; 98.2–100	100; 99.7–100
**Geenius HIV 1/2 confirmatory assay – automated reading**							
Sensitivity; 95% CI	100; 98.3–100	96.2; 77.8–99.5	100; 98.3–100	100; 98.4–100	100; 98.3–100	100; 98.3–100	99.7; 97.8–100
Specificity; 95% CI	98.4; 95.0–99.5	98.3; 94.8–99.5	97.0; 93.0–98.7	100; 98.4–100	98.9; 96.9–99.6	98.4; 95.8–99.4	98.4; 97.5–99.0
PPV; 95% CI	96.5; 89.4–98.9	89.4; 72.8–96.4	88.0; 75.4–94.6	100; 98.4–100	97.7; 93.7–99.2	86.9; 71.5–94.6	95.5; 93.0–97.1
NPV; 95% CI	100; 98.1–100	99.4; 96.0–99.9	100; 97.8–100	100; 98.4–100	100; 98.4–100	100; 98.2–100	99.9; 99.3–100
**Geenius HIV 1/2 confirmatory assay – visual reading**							
Sensitivity; 95% CI	100; 98.3–100	96.2; 77.8–99.5	100; 98.3–100	100; 98.4–100	100; 98.3–100	100; 98.3–100	99.7; 97.8–100
Specificity; 95% CI	98.2; 95.0–99.4	97.8; 94.2–99.2	94.7; 90.2–97.2	100; 98.4–100	98.9; 96.8–99.6	98.3; 95.7–99.4	97.9; 96.9–98.6
PPV; 95% CI	96.1; 89.3–98.6	86.3; 70.0–94.5	80.4; 67.9–88.9	100; 98.4–100	97.7; 93.7–99.2	86.9; 71.4–94.6	94.1; 91.4–96.0
NPV; 95% CI	100; 98.1–100	99.4; 96.0–99.9	99.8; 99.2–100	100; 98.4–100	100; 98.4–100	100; 98.1–100	99.9; 99.3–100

PPV = positive predictive value, NPV = negative predictive value.

Similar to results for the RDTs, specificities of both simple confirmatory assays varied across sites, with the lowest specificities recorded on specimens from Baraka (Table 6). Unadjusted (unweighted/crude) performance data are displayed in Additional File 2.

### False reactive results and their associated risk factors

A total of 438 specimens gave false-positive results with at least one RDT. False-positive results were associated with different factors for each of the tests, as shown by the odds ratio for false-positive results in a multivariate analysis (Table 7). For Determine, the main determinant for a false-positive result was to be referred for testing by a clinician from the IPD, OPD or the TB clinic (i.e. possibly due to presence of comorbidities), whereas with Genie Fast and Vikia, a false positive was mostly strongly associated with being male. Differences by origin remained significant only for INSTI, SD Bioline, and First Response. More detailed analyses per test are provided in Additional File 3.

**Table 7.** Odds ratio for false-positive results in a multivariate analysis for each rapid test.

		Determine	Uni-Gold	Genie Fast	Vikia	INSTI	SD Bioline	First Response
**Gender**								
	Female	Ref	Ref	Ref	Ref	Ref	Ref	Ref
	Male	1.77 (0.21–2.6)	1.57 (0.80–3.1)	**2.94 (1.90–4.6)**	**2.09 (1.22–3.6)**	**1.68 (1.17–2.4)**	0.68 (0.35–1.3)	0.88 (0.60–1.3)
**Age group**								
	<15	Ref	Ref	Ref	Ref	Ref	Ref	Ref
	15–29	0.80 (0.29–2.2)	1.16 (0.15–9.2)	1.23 (0.35–4.3)	3.22 (0.41–25.6)	0.83 (0.30–2.3)	0.65 (0.14–3.0)	2.28 (0.52–9.9)
	30–44	0.74 (0.27–2.1)	0.67 (0.08–5.8)	1.08 (0.30–3.9)	2.76 (0.34–22.2)	0.68 (0.24–1.9)	1.09 (0.23–5.0)	2.21 (0.50–9.8)
	45–60	1.11 (0.37–3.3)	1.77 (0.20–15.9)	1.27 (0.32–4.9)	4.75 (0.57–39.5)	0.93 (0.30–2.8)	0.35 (0.05–2.6)	3.08 (0.66–14.3)
	>60	1.78 (0.51–6.3)	4.11 (0.41–41.1)	2.33 (0.51–10.8)	8.37 (0.88–80.0)	1.75 (0.49–6.2)	1.27 (0.16–9.9)	1.97 (0.33–11.9)
**Site**								
	Conakry	Ref	Ref	Ref	Ref	Ref	Ref	Ref
	Kitgum	1.65 (0.82–3.3)	0.93 (0.03–24.7)	1.40 (0.37–5.3)	1.65 (0.25–10.9)	2.07 (0.52–8.2)	3.55 (0.39–32.3)	**4.20 (1.24–14.2)**
	Arua	0.29 (0.07–1.2)	1.45 (0.05–39.3)	2.68 (0.68–10.5)	2.18 (0.32–14.8)	**7.42 (1.86–29.6)**	**9.83 (1.06–91.4)**	**11.94 (3.47–41.1)**
	Homa Bay	1.14 (0.56–2.3)	0.45 (0.02–12.9)	0.69 (0.17–2.8)	1.55 (0.24–10.0)	2.06 (0.52–8.2)	6.74 (0.79–57.8)	**6.59 (2.00–21.7)**
	Baraka	0.69 (0.33–1.4)	2.58 (0.10–66.1)	1.76 (0.47–6.7)	3.08 (0.51–18.5)	**17.6 (4.7–66.5)**	**10.70 (1.18–96.9)**	3.33 (0.91–12.1)
	Douala	1.14 (0.55–2.3)	1.10 (0.04–29.0)	0.91 (0.23–3.7)	1.51 (0.23–9.8)	**9.9 (2.6–38.4)**	2.16 (0.19–24.3)	0.17 (0.02–1.75)
**Entry mode**								
	Voluntary	Ref	Ref	Ref	Ref	Ref	Ref	Ref
	Spouse	0.68 (0.15–3.0)	N/A	0.70 (0.15–3.3)	2.06 (0.39–10.8)	1.07 (0.25–4.6)	1.74 (0.24–12.5)	0.29 (0.03–2.4)
	Referred^a^	**1.79 (1.07–3.0)**	1.46 (0.63–3.4)	1.43 (0.76–2.7)	1.48 (0.70–3.2)	1.23 (0.79–1.9)	1.30 (0.50–3.4)	0.76 (0.34–1.7)
	ANC	0.50 (0.11–2.3)	0.46 (0.02–11.3)	0.81 (0.21–3.1)	N/A	2.14 (0.63–7.3)	1.50 (0.26–8.6)	1.28 (0.52–3.1)
**Comorbidity**								
	Malaria	2.62 (1.21–5.6)	1.81 (0.39–8.3)	1.19 (0.42–3.3)	1.86 (0.60–5.7)	1.00 (0.38–2.6)	1.86 (0.54–6.4)	0.97 (0.41–2.3)
	TB	1.08 (0.36–3.3)	N/A	0.54 (0.13–2.3)	0.69 (0.13–3.8)	0.84 (0.26–2.7)	N/A	1.35 (0.34–5.4)

Significant results are highlighted in bold. N/A = not applicable. STAT-PAK was excluded because too few false-positive results were obtained. ^a^Referred by a clinician from the in-patient department, the out-patient department or the TB clinic.

## Discussion

Growing awareness of problems with patient misdiagnosis at some HIV testing sites in sub-Saharan Africa, and inconsistent findings on the accuracy of widely used simple diagnostic tests, have highlighted the urgent need for a comprehensive, systematic evaluation of these tests, with special emphasis variation in their performance by geographical location and other characteristics [5]. All but one of the RDTs evaluated here has been WHO prequalified, and of them, only STAT-PAK recorded a final sensitivity of less than 100% (99.5%) [6,7]. The final specificities in the WHO prequalification evaluations were: 100% for STAT-PAK, 99.9% for SD Bioline and Vikia, 99.4% for First Response and 98.9% for Determine [6,7]. However, in our evaluation, individual RDTs performed more poorly than in WHO evaluations with only one test (STAT-PAK) meeting the recommended thresholds for RDTs of ≥99% sensitivity and ≥98% specificity when using total estimates [1]. None of the tests met the WHO-recommended thresholds for sensitivity and specificity when using the lower end of the 95% CI [1].

While all but one HIV RDT and two simple confirmatory assays had total adjusted sensitivities ≥99%, the biggest problem identified was specificity, which varied widely among the different tests and by samples’ origin. Only one of the eight tests (STAT-PAK) had a total adjusted specificity ≥98%, exceeding the WHO-recommended threshold (lower end of the 95% CI of ≥98%) [1] at five of six sites; two other tests (SD Bioline and First Response) exceeded it at one site. Although confirmatory assays are presumed to have higher specificity than RDTs, the two simple confirmatory assays evaluated here showed a specificity ≥98% at only half the study sites. None of the confirmatory assay met the WHO threshold of the lower end of the 95% CI interval of ≥99% [1].

It has been proposed that cross reactivity, either direct or indirect, may be responsible for the variable performance of RDTs in different populations and test sites, and that concomitant disease, such as kala azar, sleeping sickness and schistosomiasis, could play a role [23–26]. Polyclonal B cell activation to various infections could account for the heterogeneity in test performance across different populations [27]. In our study, co-morbidities were assessed only by self-reporting, and no significant association with false reactive results could be established.

Interestingly, being referred by a clinician from the IPD, OPD or TB clinic (as a result having one or more co-morbidities) was a risk factor for false reactivity, but only for Determine. In contrast, for Genie Fast and Vikia, the main risk factor associated with false reactive results was male gender with a 2–3-fold increased risk. Finally, the origin of the participants was highly associated with false reactivity on the INSTI, SD Bioline and First Response tests, indicating the presence of unknown site-specific factors.

It has been postulated that weak reactive test lines/dots are more likely to be false positive than true positive results and that considering them as potentially negative might reduce false-positive results [2,10,15,18,19,28,29]. We detected weak testing lines only with SD Bioline and First Response, the latter showing weak results on almost 50% of reactive tests for HIV-2. For other tests, however, no weak lines were reported, meaning that even false reactive/positive results produced a line of at least medium intensity. This presumably helped reduce variability between test readers: inter-reader agreement was very high (kappa coefficients ≥0.98) for all tests, in line with WHO recommendations of an inter-reader variability <5% [1].

Specificity for HIV-2 for the SD Bioline and First Response tests was low: 89.8% and 96.1% respectively. This confirms results of the WHO prequalification evaluations, which found that RDTs showed a wide range of cross-reactivity (3–57%) on the HIV-2 line, potentially leading to significant false diagnosis of HIV-2 infections. However, as the concerned RDTs are WHO prequalified, providers and patients may be lead to believe that they are double-infected or solely infected with HIV-2, a less aggressive form of the virus [7].

Several possible limitations related to the use of RDTs in this study should be noted. First, RDTs are designed for use on fresh specimens; in practice this typically means capillary whole blood. As it happened, this study used plasma samples that had been frozen, shipped, and stored before testing. Some studies have shown differences in sensitivity and specificity when using plasma/serum compared to capillary whole blood [13,28,30]. Second, our evaluation was carried out on one batch of index tests, precluding a comparison between batches. Third, considering the relatively low prevalence of HIV in some testing sites, we decided not to include all consecutive clients but, instead, all consecutive positives and a fixed number of negative clients. In doing so, we introduced verification bias, resulting in a sample that was not representative of the overall population. We therefore performed a weighted analysis to account for the sampling strategy, and acknowledge that these estimates are not as solid as they would be had we carried out consecutive sampling. Last but not least, the simple confirmatory assays need to be evaluated in as part of an algorithm in addition to individual performance.

## Conclusions

In summary, the findings of this large multi-centre study indicate that HIV RDT performance can vary greatly according to patient’s gender, comorbidities, and other unknown factors associated to geographic location, even within in a single country. By performing all tests in a centralized setting, we show that these differences in performance cannot be attributed to study procedure, end-user variation or storage conditions. Also, simple confirmatory assays in this study had imperfect and varying specificities according to origin of specimens, suggesting that they may not provide an appropriate universal solution in all geographical locations to the problem of false-positive results. Finally, these results underscore the need for local validation of HIV RDTs in order to design accurate testing algorithms.

## Notes

To access the supplementary material to this article please see Supplementary Files under Article Tools online.
